# 

**Published:** 1896-12

**Authors:** 


### CONTENTS—DECEMBER, 1896.—VOL. XII., NO. 6.

ORIGINAL CONTRIBUTIONS—

Something on Medical Mistakes. B. E. Hadra, M. D . . . . . . . . . . . . . . . . 289
Hereditary Insanity . . . . . . . . . . . . . . . . . . . . . . . . . . . . . . . 300
A New Method of Treating Carbuncle. T. C. Osborn, M. D . . . . . . . . . . . . 302
Typho-Malarial Fever. I. P. Sessions, M. D. . . . . . . . . . . . . . . . . . . 309
Civilization and Tuberculosis. F. Paschal, M. D . . . . . . . . . . . . . . . . 314
Richmond Academy of Medicine and Surgery . . . . . . . . . . . . . . . . . . . 316

SOCIETY NOTES—

Brazos Valley Medical Association. . . . . . . . . . . . . . . . . . . . . . . 322
South Texas Medical Society. . . . . . . . . . . . . . . . . . . . . . . . . . 323

ABSTRACTS AND SELECTIONS—

Modern Treatment of Diphtheria in Private Practice . . . . . . . . . . . . . . 324

EDITORIAL—

A Degenerate Son . . . . . . . . . . . . . . . . . . . . . . . . . . . . . . . 327
Notes on Organization . . . . . . . . . . . . . . . . . . . . . . . . . . . . . 329
Dr. Paschal's Paper . . . . . . . . . . . . . . . . . . . . . . . . . . . . . . 331
Dr. Hamilton Again . . . . . . . . . . . . . . . . . . . . . . . . . . . . . . 334
New Treatment of Carbuncle . . . . . . . . . . . . . . . . . . . . . . . . . . 335
The Best are Barred . . . . . . . . . . . . . . . . . . . . . . . . . . . . . . 335
The Doctors . . . . . . . . . . . . . . . . . . . . . . . . . . . . . . . . . . 338

CORRESPONDENCE—

Medical Legislation . . . . . . . . . . . . . . . . . . . . . . . . . . . . . . 339

MEDICAL NEWS AND MISCELLANY. . . . . . . . . . . . . . . . . . . . . . . . . . 340–342
BOOK NOTICES . . . . . . . . . . . . . . . . . . . . . . . . . . . . . . . . . . 343
PUBLISHRRS' NOTES . . . . . . . . . . . . . . . . . . . . . . . . . . . . . . . 343–350

